# External validation of predictive scores for diabetes remission after metabolic surgery

**DOI:** 10.1007/s00423-021-02260-3

**Published:** 2021-07-13

**Authors:** Izabela A. Karpińska, Joanna Choma, Michał Wysocki, Alicja Dudek, Piotr Małczak, Magdalena Szopa, Michał Pędziwiatr, Piotr Major

**Affiliations:** 1 2nd Department of General Surgery, Jagiellonian University Medical College, Jakubowskiego 2 st., 30-688, Kraków, Poland; 2 Department of General Surgery and Surgical Oncology, Ludwik Rydygier Memorial Hospital in Cracow, Kraków, Poland; 3 Kraków University Hospital, Kraków, Poland; 4 Centre for Research, Training and Innovation in Surgery (CERTAIN Surgery), Kraków, Poland; 5 Department of Metabolic Diseases, Jagiellonian University Medical College, Kraków, Poland

**Keywords:** Risk prediction scores, External validation, Diabetes remission, Type 2 diabetes mellitus, Bariatric surgery, Metabolic surgery

## Abstract

**Purpose:**

Bariatric surgery has proven to be the most efficient treatment for obesity and type 2 diabetes mellitus (T2DM). Despite detailed qualification, desirable outcome after an intervention is not achieved by every patient. Various risk prediction models of diabetes remission after metabolic surgery have been established to facilitate the decision-making process. The purpose of the study is to validate the performance of available risk prediction scores for diabetes remission a year after surgical treatment and to determine the optimal model.

**Methods:**

A retrospective analysis comprised 252 patients who underwent Roux-en-Y gastric bypass (RYGB) or sleeve gastrectomy (SG) between 2009 and 2017 and completed 1-year follow-up. The literature review revealed 5 models, which were subsequently explored in our study. Each score relationship with diabetes remission was assessed using logistic regression. Discrimination was evaluated by area under the receiver operating characteristic (AUROC) curve, whereas calibration by the Hosmer–Lemeshow test and predicted versus observed remission ratio.

**Results:**

One year after surgery, 68.7% partial and 21.8% complete diabetes remission and 53.4% excessive weight loss were observed. DiaBetter demonstrated the best predictive performance (AUROC 0.81; 95% confidence interval (CI) 0.71–0.90; p-value > 0.05 in the Hosmer–Lemeshow test; predicted-to-observed ratio 1.09). The majority of models showed acceptable discrimination power. In calibration, only the DiaBetter score did not lose goodness-of-fit in all analyzed groups.

**Conclusion:**

The DiaBetter score seems to be the most appropriate tool to predict diabetes remission after metabolic surgery since it presents adequate accuracy and is convenient to use in clinical practice. There are no accurate models to predict T2DM remission in a patient with advanced diabetes.

**Supplementary Information:**

The online version contains supplementary material available at 10.1007/s00423-021-02260-3.

## Introduction

Nowadays, the focus on bariatric treatment is gradually shifting from the primary goal of body weight reduction towards the remission of obesity-related metabolic diseases [1]. Data shows that the majority of European patients undergoing bariatric surgery has at least one comorbidity [2]. Type 2 diabetes mellitus (T2DM) is reported to be the most common, with a prevalence from 9 to 24% [2]. Surgical procedures have proven to be the most effective type of T2DM treatment, with a postoperative remission rate of up to 78% [3, 4]. These observations have recently led to changes in guidelines, which recommend bariatric procedures to be considered as “metabolic surgery” in the treatment of T2DM, even for those who are merely overweight [5].

Although an interdisciplinary group of specialists obtains a comprehensive preoperative assessment of each candidate for metabolic surgery, not all patients achieve the desirable outcome of T2DM remission [6]. Prediction of T2DM remission after surgery could be crucial for controlling diabetes. Earlier intervention may provide better long-term metabolic outcomes in patients with a high possibility of diabetes remission [5, 7]. Since surgery may also pose many complications requiring long-term monitoring and supplementation, preoperative assessment of diabetes resolution could prevent unnecessary surgical procedures and risks [8]. The ability to distinguish patients eligible for surgical treatment is also economically beneficial as it would decrease long-term healthcare costs for the entire public health system [9].

Efforts have been made to explore multiple predictors of diabetes remission after surgery, and as a result, numerous risk prediction scores were proposed and validated [10]. Still, there is no scientific consensus on the most accurate to be used in clinical practice. Considering these facts, we designed a study to compare available risk prediction models for postoperative T2DM remission and to determine the one with the best predictive accuracy and clinical applicability.

## Material and methods

### Study design and patients

In this retrospective study, we included patients with T2DM who underwent laparoscopic sleeve gastrectomy (SG) or Roux-en-Y gastric bypass (RYGB) in our hospital from April 2009 to October 2017 and completed 1 year of postoperative follow-up. Patients with preexisting severe complications of T2DM, type 1 diabetes mellitus, prior bariatric surgeries, and those who were qualified for reoperation were excluded from the study.

We divided the study population into 3 groups: *ALL group* including patients after either RYGB or SG, *RYGB group* including patients after RYGB, and *SG group* including patients after SG.

Patients undergoing bariatric surgery were evaluated by a multidisciplinary team of surgeons, diabetologists, psychologists, clinical nurse specialists, dietitian nutritionists, and anesthetists. Demographic, anthropometric, and clinical data were recorded pre- and postoperatively. The follow-up schedule comprised appointment 12 months after surgery.

Informed consent for surgical treatment was obtained from all patients before surgery. All procedures performed in the study involving human participants were in accordance with the 1964 Helsinki Declaration and its later amendments.

### Surgical techniques

All patients included in our study underwent either laparoscopic SG or laparoscopic RYGB performed by experienced surgeons. Each patient was qualified for the appropriate type of procedure in accordance with the Polish Guidelines for Metabolic and Bariatric Surgery [11]. The surgical techniques used in our department have been described in detail in our previous publications [12, 13]. The length of an alimentary and enzymatic limb during RYGB was standardized in all patients, 150 and 100 cm respectively.

### Data collection

Sex, age, height, weight, body mass index (BMI), duration of diabetes, current diabetes medications, comorbidities, micro- and macrovascular diabetic complications, and laboratory results were collected retrospectively from medical records. Duration of diabetes was defined as the difference between the date of T2DM diagnosis and the date of surgery. Diabetes medications were classified as follows: glucose-lowering medications (GLM) including glucagon-like peptide-1 (GLP-1) analogs, dipeptidyl peptidase 4 (DPP-IV) inhibitors, sulfonylureas, thiazolidinediones (TZDs), glinides, α-glucosidase inhibitors, and metformin and insulin (basal and bolus). The number of glucose-lowering agents prescribed was considered the sum of the above drug categories. Investigated comorbidities included hypertension (blood pressure > 140/90 mmHg or antihypertensive treatment), hyperlipidemia, metabolic syndrome (defined by IDF, NHLBI, AHA, WHF, IAS, and IASO criteria from 2009), liver disease, obstructive sleep apnea (OSA), polycystic ovary syndrome (PCOS), and gastroesophageal reflux disease (GERD). Microvascular complications were defined as the presence of diabetic nephropathy, retinopathy, or neuropathy, whereas macrovascular complications were defined as the presence of coronary artery disease (CAD), arteriosclerosis, stroke, or atherosclerotic acute limb ischemia. Laboratory investigations included fasting blood glucose (FBG), glycated hemoglobin (HbA1c), high-density lipoprotein (HDL), low-density lipoprotein (LDL), total cholesterol (TC), triglycerides (TG), glutamic pyruvic transferase (GPT), and aspartate transaminase (AspAT). Blood samples were collected at baseline after 12 h of overnight fasting. Percentage weight loss (%WL), percentage excess weight loss (%EWL), and percentage excess body mass index loss (%EBMIL) were chosen as the outcome measures for weight change after surgery.

### Outcome measurement

Diabetes remission was defined by the American Diabetes Association (ADA) criteria from 2009 [14]. Endpoints assessed in our analysis included complete and partial remission of T2DM. Complete remission of diabetes was defined by HbA1c < 6.0% and FBG < 5.6 mmol/L and no use of oral or injectable diabetes medication for at least 12 months. Partial remission was defined by HbA1c < 6.5% and FBG < 7.0 mmol/L and no use of oral or injectable diabetes medication for a minimum of 12 months.

### Model selection

Searches of PubMed, Embase, and Cochrane Library databases were performed on November 5, 2019. The following search terms were used: risk prediction models, bariatric or metabolic surgery, and diabetes remission. We found 7 preoperative risk prediction scores of diabetes remission after bariatric surgery. Models consisting of postoperative variables or variables not routinely checked in our daily practice were excluded. Ultimately, we selected 5 scores including individualized metabolic surgery (IMS), DiaRem, advanced DiaRem (Ad-DiaRem), DiaBetter, and the model proposed by Robert et al. [15–19].

The complete overview of preoperative variables for each model and details of scoring is provided in Online Resource 1.

### Statistical analysis

Continuous variables are presented as mean with standard deviation (SD) or median with interquartile range (IQR) for normally and non-normally distributed variables respectively.

The scores and odds of diabetes remission of five models were calculated for each patient. The score was calculated using preoperative data according to the definition of the original scoring model. The scores’ relationship with the odds of diabetes remission was assessed using the logistic regression method. Associations between the scores and diabetes remission were expressed as odds ratios (OR) with 95% confidence intervals (95% Cl). To assess the diagnostic accuracy of each model, discrimination and calibration were evaluated. To assess the discrimination of the scores, we used receiver operating characteristic (ROC) curves and the area under the ROC (AUROC) curves. Calculation comparing the AUROC of the scores was made with the use of the U-statistic originally proposed in Hanley’s algorithm [20, 21]. The calibration of the models was assessed using the Hosmer–Lemeshow goodness-of-fit test and predicted-to-observed ratio. In the Hosmer–Lemeshow test, p > 0.05 indicated good calibration. To obtain the predicted-to-observed ratio, the predicted probability of diabetes remission was calculated using logistic regression. Statistical significance was defined as p ≤ 0.05. All calculations were done with STATISTICA 13.3 software (StatSoft Inc., Tulsa, Oklahoma, USA).

## Results

### Study recruitment

**Fig. 1:**
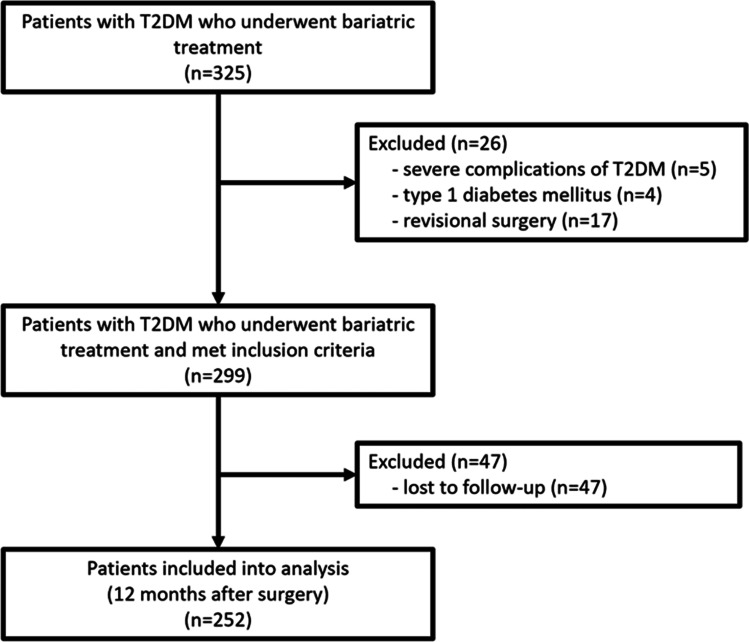
Patients flow through the study. Abbreviations: T2DM, type 2 diabetes mellitus

A total of 325 patients with T2DM underwent bariatric surgery (laparoscopic SG or laparoscopic RYGB) in our hospital from April 2009 to October 2017. Twenty-six (8%) patients were excluded because they did not meet the inclusion criteria, and 47 patients (14.46%) were excluded on account of loss to follow-up. Ultimately, the study sample comprised 252 patients (Fig. 1).

### Baseline characteristics and outcomes

Out of 252 patients enrolled in our study 150 (59.5%) were women, whereas 102 (40.5%) were men with a median age of 48 years. The most common comorbidities were hypertension (83.73%) and metabolic syndrome (80.95%). Most patients took at least one diabetes medication (85.71%) and 81 (32.14%) patients needed insulin therapy. The median diabetes duration was 6 years. One hundred eighteen (46.83%) patients underwent SG, whereas 134 (53.17%) had RYGB. Median of preoperative HbA1c was 6.75% and preoperative BMI was 45.39 kg/m^2^, both decreased to 5.8% and 33.09 kg/m^2^ respectively after 1 year, %EWL after surgery amounted to 53.4%. The differences between pre- and postoperative weight, BMI, FBG, and HbA1c were statistically significant with p < 0.0001 (Online Resource 1). The partial T2DM remission rate reached 68.7%, whereas complete remission occurred in 21.8% of patients. Detailed patient characteristics are listed in Table 1.

**Table 1:** Characteristics of the study population and postoperative results

Variable	All (n = 252)	RYGB (n = 134)	SG (n = 118)
Demographics			
Age, years	48.00 (17.00)	48.00 (16.00)	47.00 (17.00)
Height, cm	169.00 (12.00)	168.50 (12.00)	170.00 (11.00)
Weigh, kg	130.00 (26.50)	128.50 (26.00)	131.00 (29.00)
BMI, kg/m^2^	45.39 (9.31)	45.26 (7.69)	45.70 (10.23)
Gender			
Women	150 (59.52)	75 (55.97)	75 (63.56)
Men	102 (40.48)	59 (44.03)	43 (36.44)
Comorbidity			
Hypertension	211 (83.73)	113 (84.33)	98 (83.05)
SBP, mmHg	13.55 (26.00)	135.00 (26.00)	136.50 (27.00)
DBP, mmHg	85.00 (20.00)	86.00 (15.00)	84.50 (22.00)
Metabolic syndrome	204 (80.95)	103 (76.87)	101 (85.59)
Hyperlipidemia	152 (60.32)	91 (67.91)	61 (51.69)
Liver disease	47 (18.65)	27 (20.15)	20 (16.95)
OSA	60 (23.81)	34 (25.37)	26 (22.03)
PCOS	13 (5.16)	4 (2.99)	9 (7.63)
GERD	20 (7.94)	16 (11.94)	4 (3.39)
Microvascular complications	43 (17.06)	29 (21.64)	14 (11.86)
Macrovascular complications	47 (18.65)	29 (21.64)	18 (15.25)
Type of the procedure			
SG	118 (46.83)	0 (0.00)	118 (100.00)
RYGB	134 (53.17)	134 (100.00)	0 (0.00)
Diabetes characteristics			
FBG, mmol/L	7.67 (3.53)	7.71 (4.09)	7.65 (2.79)
HbA1c, %	6.75 (2.00)	6.80 (2.20)	6.50 (1.70)
Diabetes duration, years	6.00 (9.00)	5.00 (10.00)	7.00 (7.00)
Diabetes medications use	216 (85.71)	118 (88.06)	98 (83.05)
Number of diabetes medications	1.00 (1.00)	1.00 (2.00)	1.00 (1.00)
Metformin	190 (75.40)	103 (76.87)	87 (73.73)
GLM	42 (16.67)	33 (24.63)	9 (7.63)
Insulin	81 (32.14)	49 (36.57)	32 (27.12)
Laboratory data			
HDL, mmol/L	1.00 (0.35)	1.00 (1.00)	1.04 (0.40)
LDL, mmol/L	2.55 ± 0.87	2.47 ± 0.89	2.64 ± 0.84
TC, mmol/L	4.21 ± 0.89	4.17 ± 0.94	4.26 ± 0.83
TG, mmol/L	1.68 (1.03)	1.67 (1.05)	1.69 (1.00)
GPT, U/L	103.00 (149.00)	124.00 (189.00)	80.00 (115.00)
AspAT, U/L	93.00 (127.00)	106.00 (190.00)	72.00 (83.00)
Postoperative outcome at 1 year			
Weight, kg	94.00 (26.00)	95.00 (23.00)	92.00 (25.50)
BMI, kg/m^2^	33.09 (6.95)	33.74 (8.13)	32.49 (5.99)
WL, %	27.44 ± 11.30	26.49 ± 11.00	28.59 ± 11.61
EWL, %	53.35 ± 21.28	51.59 ± 20.88	55.49 ± 21.68
EBMIL, %	61.81 ± 25.53	59.68 ± 24.63	64.40 ± 26.49
HbA1c, %	5.80 (1.40)	5.85 (1.30)	5.50 (1.50)
FBG, mmol/L	5.11 (2.29)	5.61 (3.35)	4.97 (1.35)
Diabetes remission	228 (90.48)	122 (91.04)	106 (89.83)
Scoring models			
IMS	53.00 (40.00)	54.00 (31.00)	55.00 (19.00)
DiaRem	6.00 (12.00)	7.00 (11.00)	5.00 (10.00)
Ad-DiaRem	9.00 (6.00)	10.50 (7.00)	9.00 (5.00)
DiaBetter	4.00 (3.00)	5.00 (3.00)	4.00 (3.00)
Robert et al	2.00 (2.00)	2.00 (2.00)	3.00 (1.00)

Data are shown as mean ± standard deviation, median (interquartile range), or number (percentage). Abbreviations: *BMI*, body mass index; *SBP*, systolic blood pressure; *DBP*, diastolic blood pressure; *OSA*, obstructive sleep apnea; *PCOS*, polycystic ovary syndrome; *GERD*, gastroesophageal reflux disease; *SG*, sleeve gastrectomy; *RYGB*, Roux-en-Y gastric bypass; *FBG*, fasting blood glucose; *HbA1c*, glycated hemoglobin; *GLM*, glucose-lowering medications; *HDL*, high-density lipoprotein; *LDL*, low-density lipoprotein; *TC*, total cholesterol; *TG*, triglycerides; *GPT*, glutamic pyruvic transferase; *AspAT*, aspartate transaminase; *WL*, weight loss; *EWL*, excess weight loss; *EBMIL*, excess body mass index loss; *IMS*, individualized metabolic surgery; *Ad-DiaRem*, advanced DiaRem

### Predictive power and diagnostic accuracy of the scores as predictors of partial T2DM remission

**Fig. 2:**
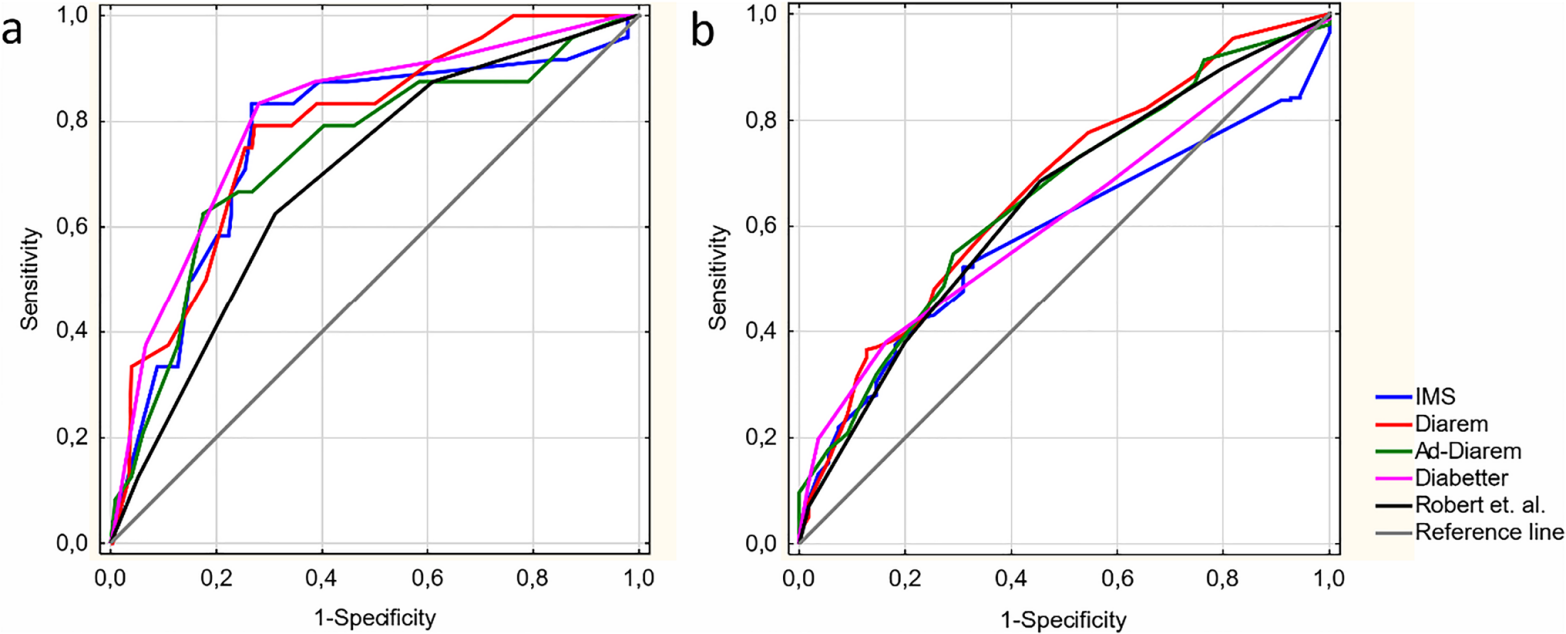
ROC curves of predictive models for partial (**a**) and complete (**b**) diabetes remission. Abbreviations: IMS, individualized metabolic surgery; Ad-DiaRem, advanced DiaRem

According to the logistic regression analysis, all scores were predictive of diabetes remission in patients after either RYGB or SG. Detailed results are shown in Table 2. Sensitivity and specificity of scoring models are demonstrated as ROC curves in Fig. 2a. In the ALL group, the DiaBetter score revealed excellent discrimination power with an AUROC of 0.81. Furthermore, it turned out to have significantly better discrimination than Robert et al., IMS, and Ad-DiaRem scores. In the RYGB group, the highest discrimination was present in Robert et al., whereas for the SG group it was the DiaRem score. Detailed results of the discrimination of each score and comparison between them are shown in Table 3 and Online Resource 1. The Hosmer–Lemeshow test results revealed that only the DiaBetter score demonstrates statistically good calibration in all three analyzed groups, whereas the IMS score was the only score which did not meet the criteria for acceptable fit in all three groups. According to predicted-to-observed ratio, most models overestimated diabetes remission from 6 to 20%. Detailed results of calibration indicators are presented in Table 4.

**Table 2:** Logistic regression for partial and complete diabetes remission in all patients, patients after RYGB and after SG

Score	All (n = 252)		RYGB (n = 134)		SG (n = 118)	
	OR (95% Cl)	p-value	OR (95% Cl)	p-value	OR (95% Cl)	p-value
Partial remission						
IMS	0.97 (0.95–0.98)	** < 0.0001**	0.97 (0.95–0.98)	**0.0003**	0.97 (0.95–0.99)	**0.009**
DiaRem	0.83 (0.77–0.90)	** < 0.0001**	0.82 (0.76–0.89)	** < 0.0001**	0.67 (0.56–0.80)	** < 0.0001**
Ad-DiaRem	0.80 (0.72–0.90)	**0.0001**	0.79 (0.71–0.87)	** < 0.0001**	0.73 (0.64–0.84)	** < 0.0001**
DiaBetter	0.51 (0.39–0.67)	** < 0.0001**	0.49 (0.37–0.64)	** < 0.0001**	0.34 (0.22–0.54)	** < 0.0001**
Robert et al	1.93 (1.25–2.98)	**0.0031**	3.54 (2.28–5.48)	** < 0.0001**	5.70 (2.92–11.30)	** < 0.0001**
Complete remission						
IMS	0.99 (0.97–1.10)	**0.04**	0.97 (0.94–0.99)	**0.004**	0.97 (0.94–0.99)	**0.02**
DiaRem	0.90 (0.85–0.95)	**0.005**	0.78 (0.70–0.88)	**0.0001**	0.60 (0.47–0.77)	**0.0007**
Ad-DiaRem	0.87 (0.80–0.95)	**0.0008**	0.75 (0.65–0.86)	**0.0001**	0.70 (0.59–0.83)	** < 0.0001**
DiaBetter	0.75 (0.62–0.92)	**0.0004**	0.47 (0.33–0.66)	** < 0.0001**	0.24 (0.10–0.54)	** < 0.0001**
Robert et al	1.57 (1.19–2.07)	**0.002**	4.29 (2.45–7.50)	** < 0.0001**	4.73 (2.27–9.85)	** < 0.0001**

p-value refers to logistic regression embolden p-values indicate a statistically significant result

Abbreviations: *RYGB*, Roux-en-Y gastric bypass; *SG*, sleeve gastrectomy; *IMS*, individualized metabolic surgery; *Ad-DiaRem*, advanced DiaRem; *OR*, odds ratio; *95% Cl*, 95% confidence interval

**Table 3:** Discrimination results in predicting partial and complete T2DM remission in all patients, patients after RYGB and after SG

Score	Partial remission		Complete remission	
	AUROC (95% Cl)	p-value	AUROC (95% Cl)	p-value
All (n = 252)				
IMS	0.76 (0.66–0.87)	** < 0.0001**	0.58 (0.51–0.66)	**0.03**
DiaRem	0.78 (0.69–0.87)	** < 0.0001**	0.61 (0.59–0.75)	**0.003**
Ad-DiaRem	0.74 (0.63–0.85)	** < 0.0001**	0.66 (0.58–0.73)	**0.0001**
DiaBetter	0.81 (0.71–0.90)	** < 0.0001**	0.67 (0.54–0.69)	** < 0.0001**
Robert et al	0.67 (0.58–0.80)	** < 0.0001**	0.64 (0.56–0.72)	**0.0007**
RYGB (n = 134)				
IMS	0.70 (0.61–0.79)	** < 0.0001**	0.70 (0.60–0.81)	**0.0001**
DiaRem	0.79 (0.71–0.87)	** < 0.0001**	0.79 (0.71–0.90)	** < 0.0001**
Ad-DiaRem	0.75 (0.67–0.83)	** < 0.0001**	0.78 (0.68–0.87)	** < 0.0001**
DiaBetter	0.79 (0.71–0.87)	** < 0.0001**	0.86 (0.70–0.88)	** < 0.0001**
Robert et al	0.82 (0.75–0.90)	** < 0.0001**	0.82 (0.79–0.93)	** < 0.0001**
SG (n = 118)				
IMS	0.66 (0.56–0.76)	**0.002**	0.68 (0.59–0.78)	**0.0001**
DiaRem	0.90 (0.84–0.96)	** < 0.0001**	0.81 (0.82–0.96)	** < 0.0001**
Ad-DiaRem	0.78 (0.70–0.86)	** < 0.0001**	0.80 (0.72–0.88)	** < 0.0001**
DiaBetter	0.82 (0.74–0.89)	** < 0.0001**	0.89 (0.74–0.90)	** < 0.0001**
Robert et al	0.82 (0.74–0.89)	** < 0.0001**	0.79 (0.70–0.88)	** < 0.0001**

Embolden p-values indicate a statistically significant result. Abbreviations: *RYGB*, Roux-en-Y gastric bypass; *SG*, sleeve gastrectomy; *IMS*, individualized metabolic surgery; *Ad-DiaRem*, advanced DiaRem; *AUROC*, area under the receiver operating characteristic; *95% Cl*, 95% confidence interval; *PPV*, positive predictive value; *NPV*, negative predictive value

**Table 4:** Calibration indicators in predicting partial and complete T2DM remission in all patients, patients after RYGB and after SG

Score	All (n = 252)		RYGB (n = 134)		SG (n = 118)	
	Hosmer–Lemeshow test	Predicted-to-observed ratio	Hosmer–Lemeshow test	Predicted-to-observed ratio	Hosmer–Lemeshow test	Predicted-to-observed ratio
Partial remission						
IMS	0.03	1.10	0.003	1.13	< 0.0001	0.40
DiaRem	**0.76**	1.11	**0.14**	1.17	0.004	1.06
Ad-DiaRem	**0.24**	1.11	**0.41**	0.83	0.001	0.90
DiaBetter	**0.64**	1.09	**0.06**	0.91	**0.36**	1.08
Robert et al	**0.95**	1.11	0.03	0.89	**0.44**	1.20
Complete remission						
IMS	0.05	1.05	0.06	1.03	0.0001	0.98
DiaRem	**0.45**	1.00	**0.36**	1.11	0.02	1.00
Ad-DiaRem	**0.62**	1.04	**0.69**	1.07	0.04	1.21
DiaBetter	**0.46**	0.94	**0.13**	0.91	**0.65**	0.98
Robert et al	**0.73**	0.99	**0.68**	1.00	**0.72**	0.90

Results of the Hosmer–Lemeshow test are shown as p-value embolden p-values indicate good calibration. Abbreviations: *RYGB*, Roux-en-Y gastric bypass; *SG*, sleeve gastrectomy; *IMS*, individualized metabolic surgery; *Ad-DiaRem*, advanced DiaRem

### Predictive power and diagnostic accuracy of the scores as predictors of complete T2DM remission

In logistic regression, all models were predictive for complete T2DM remission after surgery in three analyzed groups (Table 2). Sensitivity and specificity of scoring models are demonstrated as ROC curves in Fig. 2b. In three analyzed groups: ALL, RYGB, and SG, the DiaBetter score presented the highest discrimination power with AUROC equal to 0.67, 0.86, and 0.89, respectively. Detailed results of the discrimination of each score and statistical comparison between them are shown in Table 3 and Online Resource 1, respectively. In calibration analysis, only DiaBetter and Robert’s scores did not lose their goodness-of-fit in all examined groups. The Predicted-to-observed ratio shows that IMS, DiaRem, and Ad-DiaRem tend to overestimate the outcome from 3 to 21%, whereas DiaBetter and Robert’s scores tend to underestimate the outcome from 1 to 10%. Detailed results of calibration indicators are presented in Table 4.

## Discussion

Our findings confirmed a well-proven statement that bariatric surgery is an effective method of obesity and T2DM treatment [3, 4, 9, 22–26]. Implemented procedures resulted in significant postoperative weight loss and BMI reduction. More importantly, they showed beneficial effects on T2DM improvement with a significant decline in FBG and HbA1c.

Our study demonstrates that decreasing IMS, DiaRem, Ad-DiaRem, and DiaBetter scores, and increasing score proposed by Robert et al. were significantly associated with increasing likelihood of diabetes remission 1 year after bariatric surgery. The majority of scales presented at least acceptable discrimination. Only the DiaBetter score presented good calibration in all analyzed groups.

The overall diabetes remission rate in our study reached 90.5% which is far higher than those reported by other authors. Yu et al. pointed that 73.5% of the study population acquired alleviation of T2DM [27]. Nonetheless, the baseline BMI level in mentioned research was prominently lower compared to our analysis. This may be the reason for the observed difference as some pieces of evidence suggest that patients with higher BMI are more likely to gain diabetes remission [28]. In another study, Shen et al. reported an 80.5% T2DM remission rate [29]. However, patients included in the analysis underwent exclusively SG, which according to recent data seems to achieve lower rates of diabetes remission [30–32].

Based on our findings, we aimed to provide the most comprehensive external validation of current risk prediction models of diabetes remission 1 year after bariatric surgery and identify the optimal one to use in clinical practice. There were some attempts in the literature to provide such a comparison. Shen et al. performed a similar evaluation of scoring and logistic regression models with patients strictly after SG and Kam et al. who compared the performance of four risk scores for diabetes remission after RYGB [29, 33]. As both of these analyses were done on Asian populations which tend to have higher diabetes prevalence with increase insulin resistance despite a lower BMI, their findings cannot be easily extrapolated to the worldwide population. The abovementioned researches focused on one type of surgical procedure. Our study comprised patients after RYGB or SG, the two most frequently performed bariatric surgeries in equal proportion [34]. Hence, it could provide more reliable pieces of evidence in the utility of risk scores in clinical practice.

The IMS score categorizes T2DM into 3 validated stages of severity. The authors went a step further and provided recommendations on procedure selection based on the risk–benefit ratio. Patients with more severe T2DM achieved lower T2DM remission rates. However, the prediction properties of the scale were not reported in the original research [15]. In our study, IMS reaches acceptable discrimination with an AUROC value of 0.76, but its estimation differs greatly from the actual condition illustrated with the Hosmer–Lemeshow test in the majority of studied groups (p-value from < 0.001 to 0.05). Previous study externally validating IMS presented better discrimination power of the score with AUROC equal to 0.85 but had the same results according to calibration [29]. Observed differences may stem from differences in ethnic characteristics of the study group.

The DiaRem score was proposed by Still et al. and validated in several subsequent studies [17, 18, 35]. Using cutoff points of 7–8, the authors established excellent discrimination with AUROC from 0.84 to 0.87. In the present study, a higher cutoff point determined at 10, reduced discrimination power to acceptable (AUROC = 0.78). One of the possible explanations for the poorer performance of DiaRem in our analysis is the difference in a surgical procedure. The majority of mentioned studies investigated patients after RYGB, whereas our cohort included both RYGB and SG. The results of the study conducted by Wood et al. suggest higher discrimination power of DiaRem when evaluating patients after RYGB compared to those after SG (AUROC 0.86 vs 0.71) [36]. Interestingly, our analysis revealed strikingly different outcomes (AUROC 0.79 vs 0.90).

The Ad-DiaRem score was created based on DiaRem by adding two clinical variables and modifying values for each category to improve predictive performance [17]. In the original derivation, the Ad-DiaRem score presented excellent discrimination (AUROC = 0.91) [17]. Our analysis revealed only acceptable discrimination (AUROC = 0.74), which is comparable to the results obtained by Kam et al. (AUROC = 0.75) [33]. Moreover, the authors of the Ad-DiaRem score presented that it is significantly better in predicting T2DM remission than DiaRem in internal and external validation conducted on the French population (AUROC 0.91 vs 0.86 and 0.94 vs 0.89, respectively) [17]. In other studies, Ad-DiaRem provided a modest improvement of DiaRem predictive ability, which did not reach statistical significance [29, 37]. The present study finds the comparable performance of DiaRem and Ad-DiaRem scores among patients after RYGB; however, DiaRem seems to be more accurate than Ad-DiaRem when it comes to patients after SG.

DiaBetter is the only score established in the cohort including both RYGB and SG [18]. In the original study, DiaBetter reached excellent discrimination, similarly to DiaRem score (0.87 vs 0.87, p = 0.86) [18]. External validations of the score confirmed its excellent accuracy in predicting T2DM remission 1 year after SG and 3 years after RYGB [29, 33]. This finding stays consistent with the present study showing the AUROC value of the DiaBetter score at the level of 0.81 and no significant variation in the performance compared to the DiaRem score.

The Scoring system proposed by Robert et al. in 2013 operates mainly with markers of β-cell failure [19]. In the primary study, it presented the highest AUROC value recognized as outstanding discrimination [19]. Nevertheless, it differs greatly from our results, pointing to the worst discrimination when analyzing all study populations (AUROC = 0.67). Similarly, AUROC below 0.7 was obtained in its external validation [29]. Shen et al. suggested such poor performance may result from an unusual point-scoring algorithm [29]. Unlike others, Robert et al. proposed a model using only binary evaluation of each parameter in the scale, which could not sufficiently weigh different degrees of diabetes severity [19]. Interestingly, it presented far higher discrimination in groups after RYGB or SG exclusively.

In our analysis, we focused on partial T2DM remission analysis, because according to ADA criteria, patients with sub-diabetic hyperglycemia who achieved a steady state without treatment meet the definition of diabetes remission as their secretory reserves of β-cells could maintain FBG below the diabetic threshold [14]. More importantly, application of such criteria was necessary to provide validation comparable with previous outcomes, as a majority of examined scores were developed based on partial diabetes remission definition. However, considering both complete and partial remission as positive outcomes led to overestimated diabetes remission rate. Therefore, we provided additional analysis for the complete remission of T2DM to present fully representative results. Although all scoring systems were able to predict the complete remission of T2DM, the discrimination power decreased greatly in all cases. The DiaBetter score presented the best discrimination power in every analyzed group. Interestingly, the AUROC of this score was much higher in patients after a particular procedure than in the general population, even though the DiaBetter score is the only score in our paper which originally was developed in a cohort including both RYGB and SG procedures [18].

Additionally, the abovementioned models were assessed as the predictors of T2DM remission on patients with poorly controlled T2DM, requiring insulin therapy. According to this calculation, only DiaBetter score had the statistically significant ability to predict partial T2DM remission (OR = 0.60; p-value = 0.04). This finding indicates it as the most accurate tool. However, obtained discrimination power was unsatisfactory with AUROC 0.65 (p-value = 0.046). There were no scores with correlation to complete remission of T2DM in logistic regression. This is an important additional finding from our analysis which indicates that there are currently no accurate models to predict diabetes remission in the group of patients who should benefit from the metabolic surgery the most. On the other hand, it confirms that DiaBetter is a promising predictive tool, which have predictive potential even for such specific groups of patients.

As we are aware of the importance of long-term outcomes prediction, we conducted the 5-year follow-up analysis as well. According to 5-year observation, partial remission of T2DM was predicted by IMS and DiaBetter scores (OR 0.96 and 0.52, respectively) with acceptable discrimination power (AUROC 0.73 and 0.76, respectively). Interestingly, the DiaBetter score still had slightly higher discrimination than the IMS score even though it was originally designed to predict long-term diabetes remission outcomes. On the other hand, complete remission could be predicted only by the score proposed by Robert et al. (OR 2.40) with acceptable discrimination as well (AUROC 0.71). However, our results are biased by considerable lost-to-follow-up, at the level of 73%, and as a result low number of patients eligible to include for the analysis. Further prospective studies should be done to investigate long-term outcomes.

Effective risk models should not only provide accurate prediction but also easy application in clinical practice. The most difficult to calculate is the IMS score, mainly due to the fact that each year of diabetes duration corresponds to the different amount of points from 0 to 100. Thus, getting the total score for the patient requires a specially designed online calculator. Nonetheless, we should remember it not only predicts diabetes remission but also provides clinicians with guidance in procedure type selection. One study reported that DiaRem performance differs according to various ethnic groups [38]. Therefore, its implementation in general practice may be restricted. DiaBetter score uses only three common elements, which can be easily obtained from patients’ medical records. Although the score proposed by Robert et al. contains as many as five parameters, all of them are dichotomic. As a result, the final score can be easily calculated during patients’ assessment. In conclusion, when considering the clinical application, the DiaBetter score and score proposed by Robert et al. are the easiest to implement into day-to-day medical practice.

Based on prediction properties and clinical utility, we aimed at determining the most adequate scoring system predicting T2DM remission. The DiaBetter had one of the highest AUROC value, recognized as excellent discrimination. Moreover, it was the only score which presented good calibration in all analyzed groups of patients. Taking these findings into consideration, we may claim that DiaBetter is the best model for predicting diabetes remission at 1 year after both RYGB and SG. Additionally, the DiaBetter score is easy to calculate in clinical practice. Thus, DiaBetter is believed to facilitate the decision-making process in qualifying patients for bariatric or metabolic surgery.

### Limitations

The study has several limitations. Firstly, it has limitations inherent to single-center study and retrospective design. Although the size of our study group was relatively small, the study was adequately powered to provide reliable external validation. Furthermore, our study comprised only Caucasian patients. It is unclear whether similar findings can be transmitted to the worldwide population. Moreover, the duration of diabetes tends to be understated in retrospective analysis which may affect the overall performance of models. Secondly, we were not able to provide reliable long-term results due to significant lost-to-follow-up at 5 years after the surgery. Finally, we were unable to analyze all reported scoring systems including ABCD and DRS due to the inclusion of biomarkers not routinely measured in our department such as C-peptide and stimulated C-peptide respectively [39, 40]. However, since these scores rely on less conventional parameters, not assessed in the majority of hospitals, they presumably could not be easily implemented in clinical practice.

## Conclusion

To sum up, our study revealed the DiaBetter score to be an adequate scoring system predicting T2DM remission at 1 year after bariatric surgery. This tool displayed excellent accuracy and advantages of easy clinical application. The effectiveness of its performance remains to be warranted in further prospective researches including a larger and more diverse cohort with at least a 5-year follow-up. Additionally, it is worth emphasizing that there are no accurate models to predict T2DM remission in patients with advanced stages of the disease which indicates an emerging field for research.

## Supplementary Information

Below is the link to the electronic supplementary material.

Supplementary file1 (DOCX 42 KB)
