# Supplementary material for: External validation of predictive scores for diabetes remission after metabolic surgery
Source: Langenbecks Arch Surg. 2021 Jul 13;407(1):131–41. doi: 10.1007/s00423-021-02260-3 (PMC8847237; doi:10.1007/s00423-021-02260-3)
Supplement: Supplementary file 1 — Supplementary file1 (DOCX 42 KB) [file 423_2021_2260_MOESM1_ESM.docx]

Table 1 The overview of parameters in each score and scoring systems

| **IMS** | | |
| --- | --- | --- |
| parameter | | points |
| number of diabetes medications | 0 | 0 |
|  | 1 | 13 |
|  | 2 | 25 |
|  | 3 | 38 |
|  | 4 | 50 |
|  | 5 | 63 |
| preoperative insulin use | yes | 18 |
|  | no | 0 |
| preoperative duration of diabetes (years) | 0 | 0 |
|  | 1 | 5.6 |
|  | 2 | 11.2 |
|  | 3 | 16.8 |
|  | 4 | 22.4 |
|  | 5 | 28 |
|  | 6 | 32 |
|  | 7 | 36 |
|  | 8 | 40 |
|  | 9 | 44 |
|  | 10 | 48 |
|  | 15 | 58 |
|  | 20 | 66.4 |
|  | 25 | 74.8 |
|  | 30 | 83.2 |
|  | 35 | 91.6 |
|  | 40 | 100 |
| preoperative glycemic control | yes | 0 |
|  | no | 16 |
| **MAX score** | | **200** |
| **DiaRem** | | |
| parameter | | points |
| age (years) | <40 | 0 |
|  | 40-49 | 1 |
|  | 50-59 | 2 |
|  | >60 | 3 |
| HbA1c (%) | <6.5 | 0 |
|  | 6.5-6.9 | 2 |
|  | 7.0-8.9 | 4 |
|  | >9.0 | 6 |
| diabetes medication | other than sulfonylureas or ISA | 0 |
|  | sulfonylureas or ISA | 3 |
| insulin use | yes | 10 |
|  | no | 0 |
| **MAX score** | | **22** |
| **Ad-DiaRem** | | |
| parameter | | points |
| age (years) | 15-41 | 0 |
|  | 42-52 | 3 |
|  | 53-69 | 5 |
| HbA1c (%) | 4.5-6.9 | 0 |
|  | 7.0-7.4 | 2 |
|  | 7.5-18.4 | 4 |
| insulin use | yes | 3 |
|  | no | 0 |
| glucose-lowering agents | yes | 1 |
|  | no | 0 |
| number of glucose-lowering drugs | 0 | 0 |
|  | 1 | 1 |
|  | 2 | 2 |
|  | ≥3 | 3 |
| duration of diabetes (years) | 0-6.9 | 0 |
|  | 7.0-13.9 | 3 |
|  | ≥14 | 5 |
| **MAX score** | | **21** |
| **DiaBetter** | | |
| parameter | | points |
| HbA1c (%) | ≤6.5 | 0 |
|  | 6.6-7.2 | 1 |
|  | 7.3-8.4 | 2 |
|  | ≥8.5 | 3 |
| duration of diabetes (years) | ≤2 | 0 |
|  | 2.1-5.0 | 1 |
|  | 5.1-10.0 | 2 |
|  | ≥10.1 | 3 |
| anti-diabetic medications | 0 | 0 |
|  | metformin | 1 |
|  | other than metformin or insulin | 2 |
|  | insulin | 3 |
| **MAX score** | | **9** |
| **Robert et. al.** | | |
| parameter | | points |
| BMI ≤50 kg/m^2^ | yes | 1 |
|  | no | 0 |
| duration of diabetes (years) | 0-4 | 1 |
|  | >4 | 0 |
| HbA1c (%) | ≤7.1 | 1 |
|  | >7.1 | 0 |
| FBG (mg/dL) | ≤114 | 1 |
|  | >114 | 0 |
| OAD treatment without insulin | yes | 1 |
|  | no | 0 |
| **MAX score** | | **5** |

Abbreviations: IMS, Individualized Metabolic Surgery; HbA1c, glycated hemoglobin; Ad-DiaRem, Advanced DiaRem; BMI, body mass index; FBG, fasting blood glucose; OAD, oral antidiabetic drugs;

Table 2 Comparison between pre- and postoperative characteristic of the study population

| Parameter | Preoperative | Postoperative | p-value |
| --- | --- | --- | --- |
| Weight, kg | 130.00 (26.50) | 94.00 (26.00) | **<0.0001** |
| BMI, kg/m^2^ | 45.39 (9.31) | 33.09 (6.95) | **<0.0001** |
| FBG, mmol/L | 7.67 (3.53) | 5.11 (2.29) | **<0.0001** |
| HbA1c, % | 6.75 (2.00) | 5.80 (1.40) | **<0.0001** |

Data are shown as median (interquartile range)
p-values refer to the comparison between parameters assessed pre- and postoperatively with the use of Wilcoxon test
embolden p-values indicate statistically significant result
Abbreviations: BMI, body mass index; FBG, fasting blood glucose; HbA1c, glycated hemoglobin;

Table 3 Comparison of AUROC between different scores in all patients, patients after RYGB and after SG for partial diabetes remission

|  | IMS | DiaRem | Ad-DiaRem | DiaBetter | Robert et. |
| --- | --- | --- | --- | --- | --- |
| IMS |  | 0.46/**0.008**/**<0.0001** | 0.40/0.15/**0.006** | **0.03**/**0.006**/**<0.0001** | **0.05**/**0.004**/**0.0007** |
| DiaRem | 0.46/**0.008**/**<0.0001** |  | 0.10/0.07/**0.0001** | 0.30/0.75/**0.01** | **0.002**/0.39/**0.02** |
| Ad-DiaRem | 0.40/0.15/**0.006** | 0.10/0.07/**0.0001** |  | **0.01**/0.17/0.37 | 0.15/0.06/0.40 |
| DiaBetter | **0.03**/**0.006**/**<0.0001** | 0.30/0.75/**0.01** | **0.01**/0.17/0.37 |  | **0.001**/0.23/1.00 |
| Robert et. al. | **0.05**/**0.004**/**0.0007** | **0.002**/0.39/**0.02** | 0.15/0.06/0.40 | **0.001**/0.23/1.00 |  |

Data are shown as p-value in all study population/patients after RYGB/patients after SG
embolden p-values indicate statistically significant result
Abbreviations: RYGB, Roux-en-Y gastric bypass; SG, sleeve gastrectomy; IMS, Individualized Metabolic Surgery; Ad-DiaRem, Advanced DiaRem;

Table 4 Comparison of AUROC between different scores in all patients, patients after RYGB and after SG for complete diabetes remission

|  | IMS | DiaRem | Ad-DiaRem | DiaBetter | Robert et. |
| --- | --- | --- | --- | --- | --- |
| IMS |  | **0.003/0.02/0.0001** | **0.01**/0.06/**0.03** | 0.15/**0.03**/**<0.0001** | 0.12/**0.0008**/0.05 |
| DiaRem | **0.003/0.02/0.0001** |  | 0.51/0.30/**0.01** | **0.01**/0.54/0.06 | 0.35/0.17/**0.03** |
| Ad-DiaRem | **0.01**/0.06/**0.03** | 0.51/0.30/**0.01** |  | 0.15/0.60/0.62 | 0.69/**0.04**/0.86 |
| DiaBetter | 0.15/**0.03**/**<0.0001** | **0.01**/0.54/0.06 | 0.15/0.60/0.62 |  | 0.39/**0.03**/0.51 |
| Robert et. al. | 0.12/**0.0008**/0.05 | 0.35/0.17/**0.03** | 0.69/**0.04**/0.86 | 0.39/**0.03**/0.51 |  |

Data are shown as p-value in all study population/patients after RYGB/patients after SG
embolden p-values indicate statistically significant result
Abbreviations: RYGB, Roux-en-Y gastric bypass; SG, sleeve gastrectomy; IMS, Individualized Metabolic Surgery; Ad-DiaRem, Advanced DiaRem;
